# Supplementary material for: Endophytic Bacterium Flexivirga meconopsidis sp. nov. with Plant Growth-Promoting Function, Isolated from the Seeds of Meconopsis integrifolia
Source: Microorganisms. 2023 Nov 30;11(12):2899. doi: 10.3390/microorganisms11122899 (PMC10745605; doi:10.3390/microorganisms11122899)
Supplement: Supplementary file 1 [file microorganisms-11-02899-s001.zip › microorganisms-2692609-supplementary.pdf]

Supplementary Materials

Endophytic Bacterium *Flexivirga meconopsidis* sp. nov. with Plant Growth-Promoting Function, Isolated from the Seeds of *Meconopsis integrifolia*

Yongtao Kan [1,2,3,†], Li Zhang [4,†], Yan Wang [2,3,5], Qingyun Ma [2,3,6], Yiqing Zhou [2,3], Xu Jiang [2,3], Wei Zhang [1,*] and Zhiyong Ruan [2,3,*]

1 College of Life Sciences, Xinjiang Normal University, Urumqi 830017, China; kyt3117428630@126.com
2 State Key Laboratory of Efficient Utilization of Arid and Semi-Arid Arable Land in Northern China, Institute of Agricultural Resources and Regional Planning, Chinese Academy of Agricultural Sciences, Beijing 100081, China; a18301335070@163.com (Y.W.); mqy@webmail.hzau.edu.cn (Q.M.); zhouyiqing@caas.cn (Y.Z.); jiangxu@caas.cn (X.J.)
3 CAAS-CIAT Joint Laboratory in Advanced Technologies for Sustainable Agriculture, Institute of Agricultural Resources and Regional Planning, Chinese Academy of Agricultural Sciences, Beijing 100081, China
4 College of Life Sciences, Yantai University, Yantai 264005, China; wkiaazl@outlook.com
5 College of Resources and Environment, Tibet Agricultural and Animal Husbandry University, Linzhi 860000, China
6 National Key Laboratory of Agricultural Microbiology, Huazhong Agricultural University, Wuhan 430070, China
* Correspondence: 107622009010029@xjnu.edu.cn (W.Z.); ruanzhiyong@caas.cn (Z.R.)
† These authors contributed equally to this work.

Table S1. Average nucleotide identity (ANI) and digital DNA–DNA hybridization (dDDH) values between the genome sequence of strain Q11^T and those of closely related strains.

Reference strains	ANI (%)	dDDH (%)
Flexivirga aerilata ID2601S^T	88.82	36.20
F. caeni BO-16^T	77.42	21.00
F. endophytica KCTC 39536^T	77.24	21.50
F. oryzae R1^T	78.48	22.70

Table S2. Statistics of the protein identification results.

Name	Total spectra	Matched spectrum	Peptide	Identified protein
Supernatant	81,106	1,614	794	245
Cell	80,196	41,661	20,413	2,401

Table S4. Data of IAA production and nitrogenase activity in strain Q11^T.

Strain	IAA (mg/L)	Nitrogenase (IU/L)
Q11^T	3.26	181.80

Table S5. Physiochemical properties of the soil.

pH	SOM (g/kg)	TN (g/kg)	TK (mg/kg)	AP (mg/kg)	NH_4^+-N (mg/kg)	NO_3^-N (mg/kg)
6.01±0.11	26.45±0.86	1.44±0.03	137.51±5.76	79.73±5.61	25.92±3.89	56.79±3.66

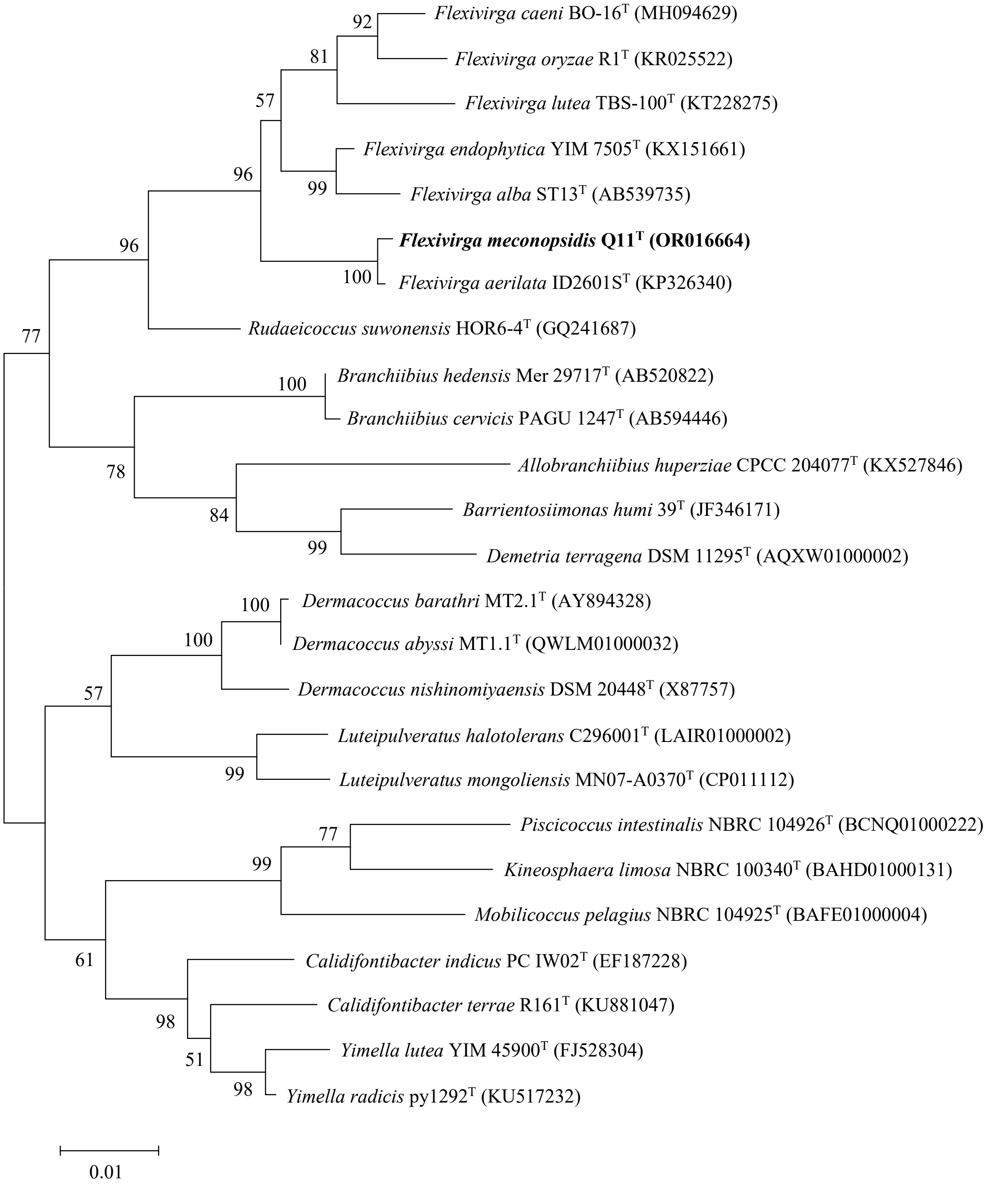

Figure S1. Maximum-likelihood phylogenetic tree of strain Q11^T and its relatives based on the comparison of the 16S rRNA gene sequences. Genbank accession numbers are shown in parentheses.

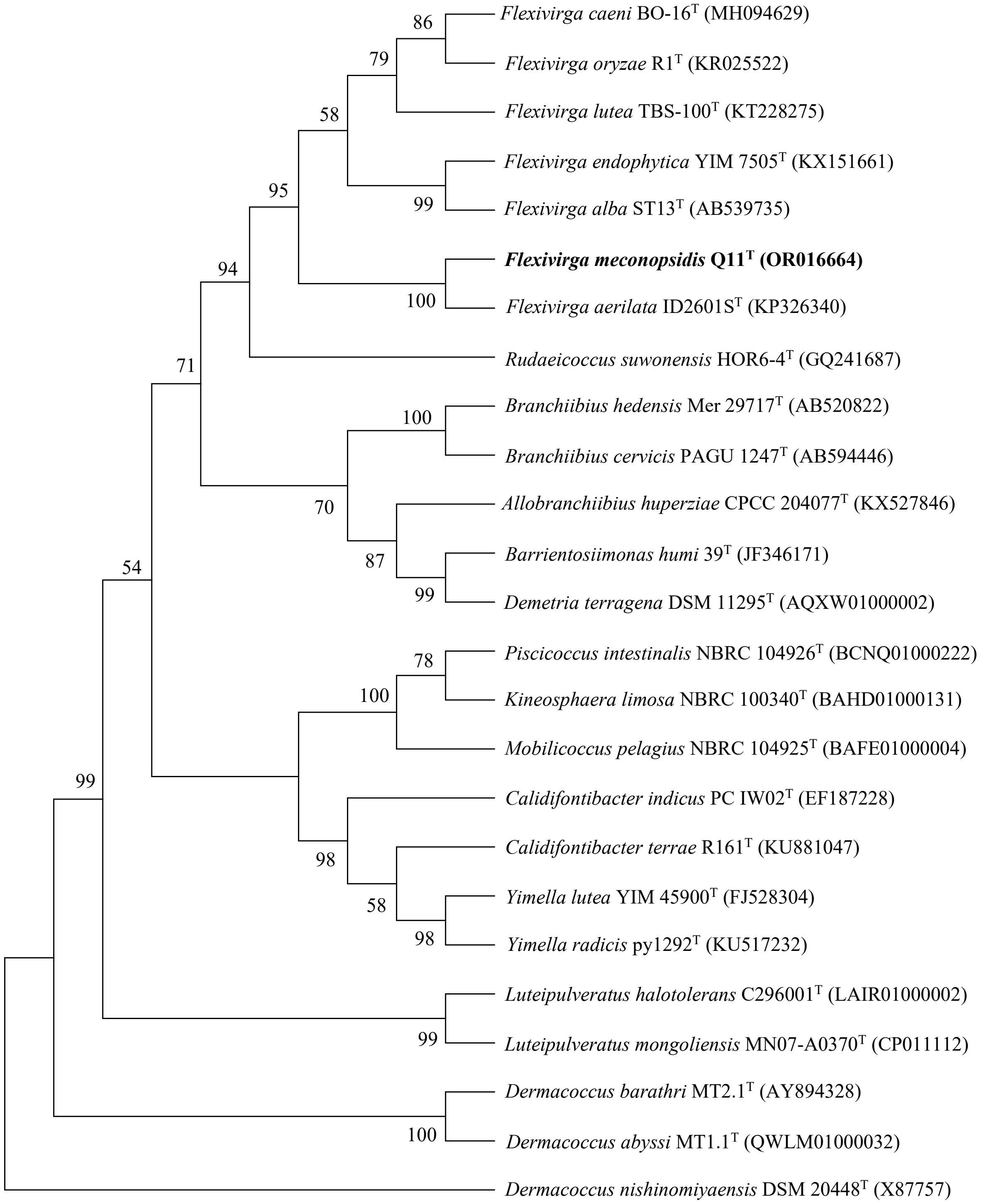

Figure S2. Maximum-parsimony phylogenetic tree of strain Q11^T and its relatives based on the comparison of the 16S rRNA gene sequences. Genbank accession numbers are shown in parentheses.

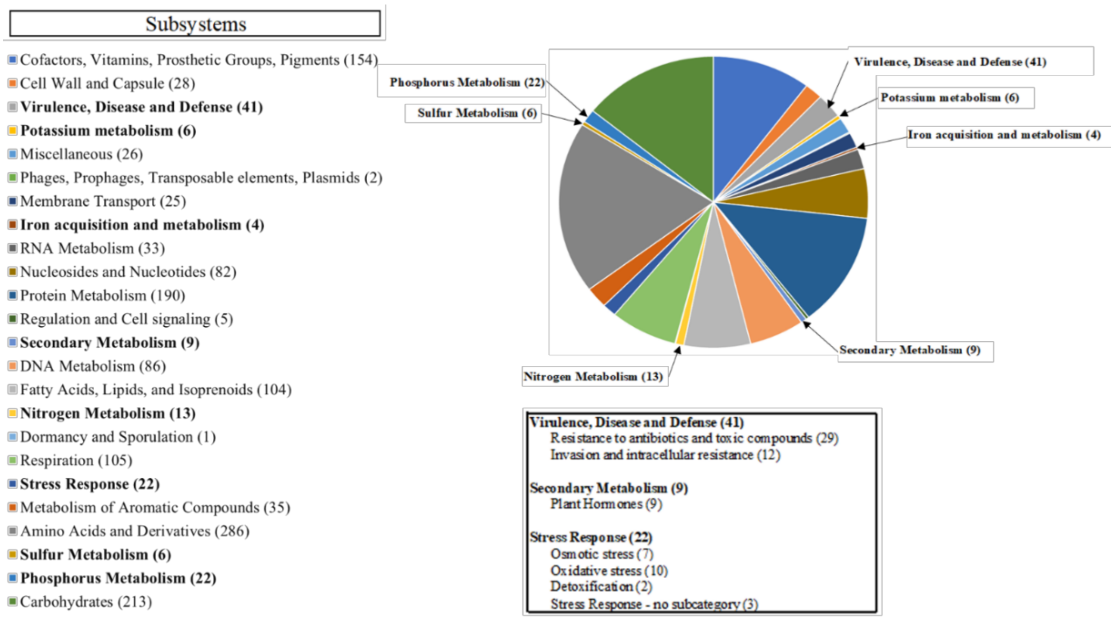

Figure S3. Different types of subsystems in the Q11^{T} genome. The data were obtained from the RAST server.

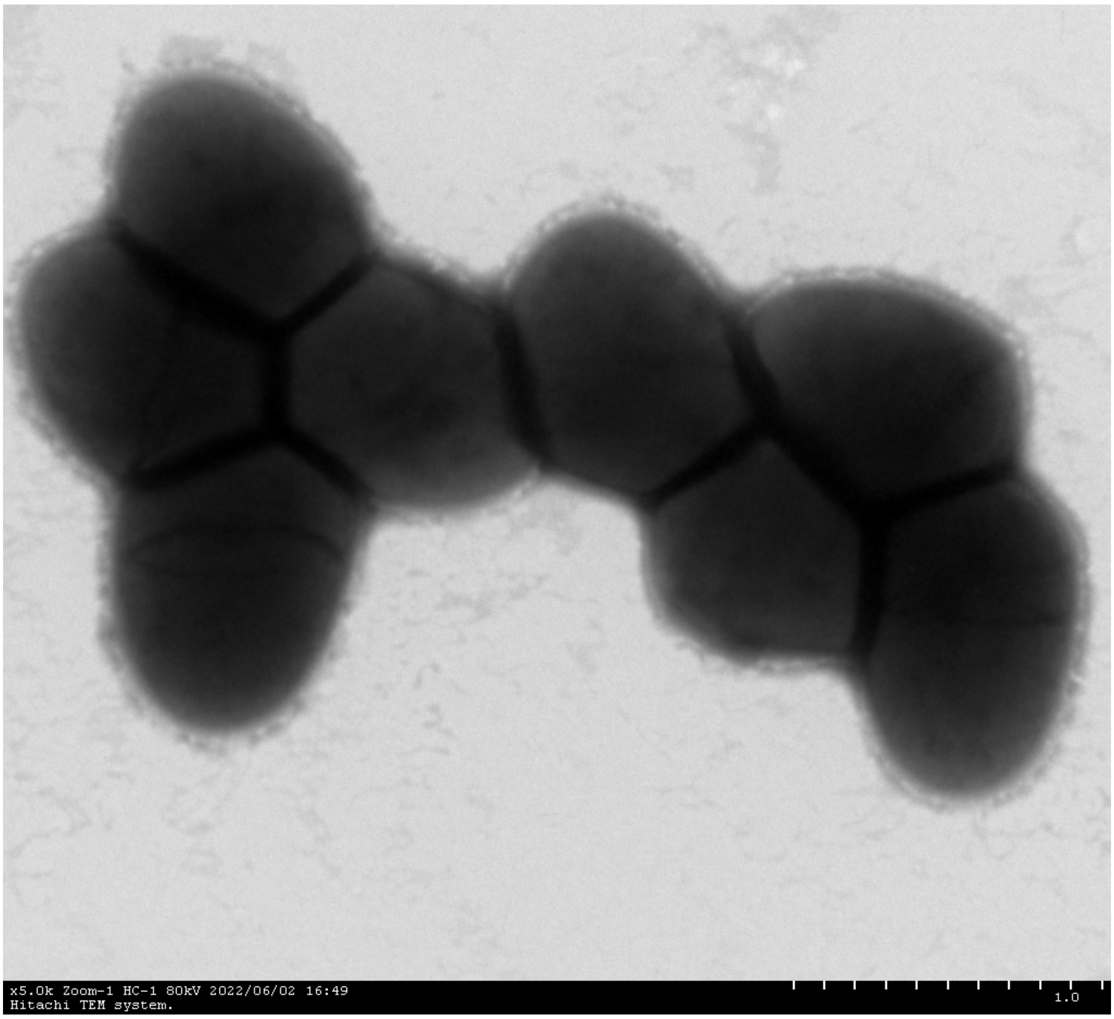

Figure S4. Transmission electron micrograph of cells of strain Q11^{T}. Bar, 1.0 μm. The cells were incubated on BA at 30 °C for 3 days.

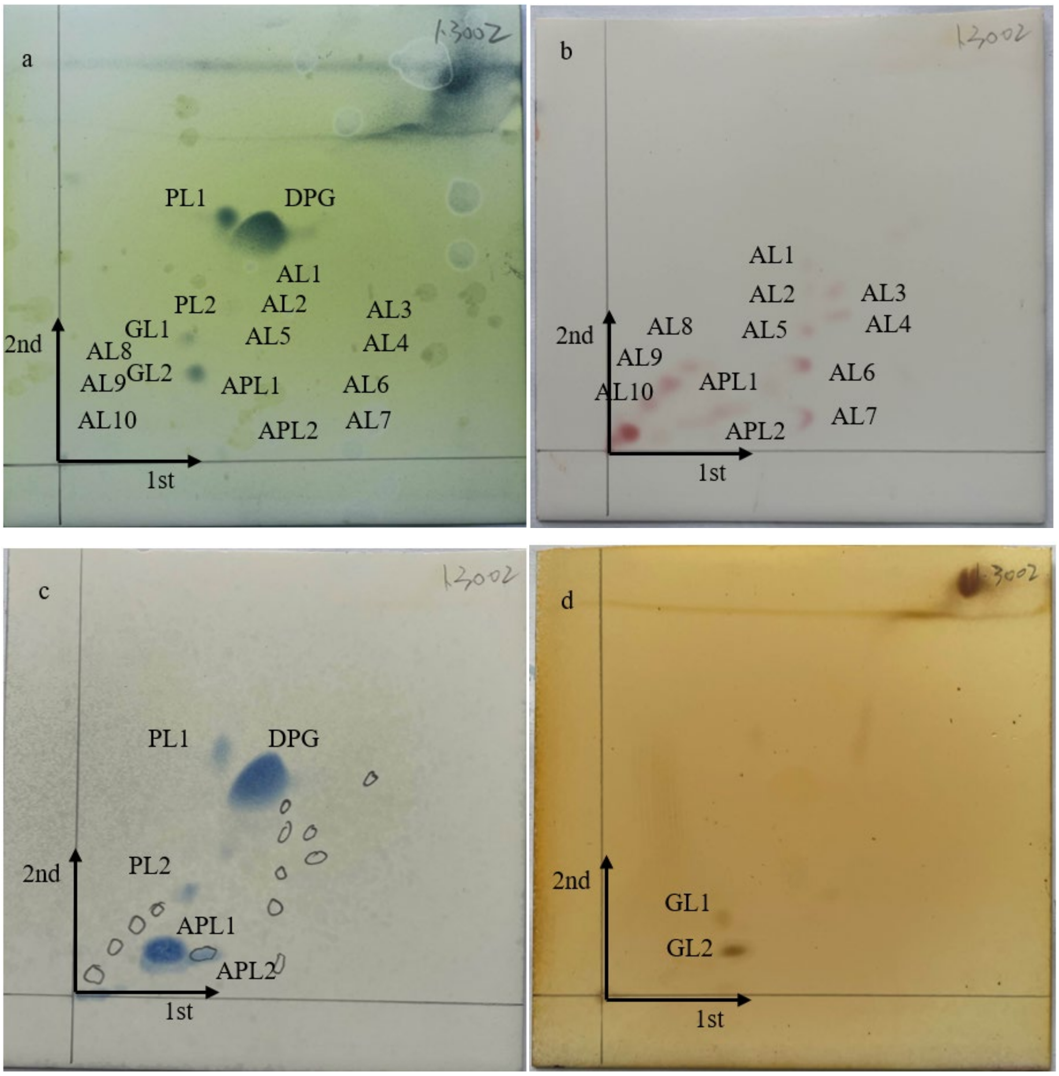

Figure S5. The polar lipids of strain Q11^{T}. Polar lipids in the strain, separated by two-dimensional TLC, which were detected by spraying with molybdatophosphoric acid. a, Molybdophosphoric acid was used to reveal the total polar lipids; b, ninhydrin was used for aminolipids; c, ammonium molybdate was used for phospholipids; and d, a-naphthol was used for glycolipids. DPG, diphosphatidylglycerol; PL (1-2), unidentified phospholipids; APL (1-2), unidentified aminophospholipid; AL (1-10), unidentified aminolipids; GL (1-2), unidentified glycolipids.

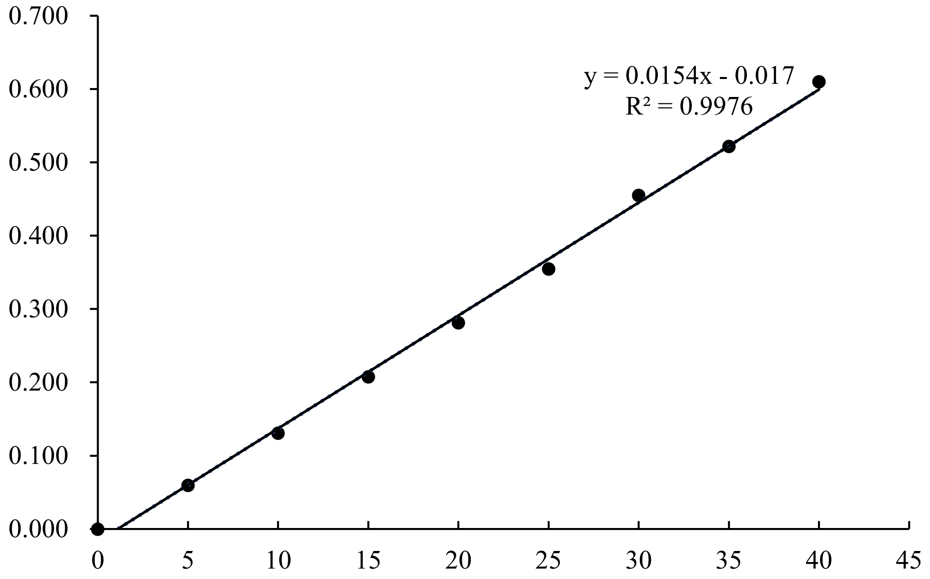

Figure S6. The indole acetic acid (IAA) standard curve.

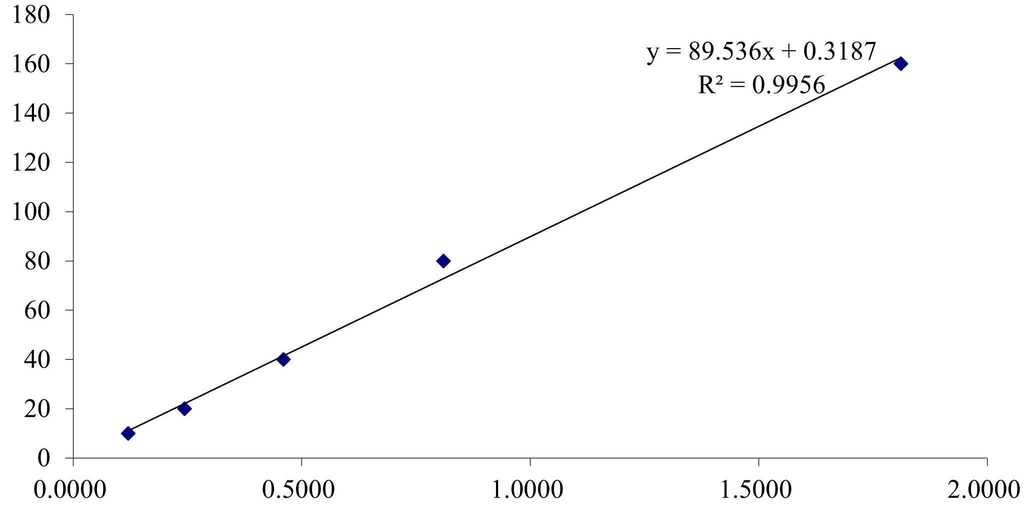

Figure S7. Standard curve of nitrogenase activity.
